# Experimental Investigation on the Fracture Behavior of PET-Modified Engineered High-Ductility Concrete: Effects of PET Powder and Precursor Composition

**DOI:** 10.3390/ma18092132

**Published:** 2025-05-06

**Authors:** Fei Meng, Shen Luo, Jingxian Sun, Cheng Zhang, Leilei Xu, Liqun Zhang, Fumin Qing, Junfeng Zeng, Ruihao Luo, Yongchang Guo

**Affiliations:** 1 Zhongshan Power Supply Bureau of Guangdong Power Grid Co., Ltd., Zhongshan 528400, China; blackstar_002@sina.cn (F.M.); zsluozong@163.com (S.L.); 1290025427@163.com (J.S.); 314355@163.com (C.Z.); 13924971515@139.com (L.X.); 13560600262@163.com (L.Z.); 2 Fujian Southeast Design Group Co., Ltd., Sanming 365000, China; supervisionr@outlook.com; 3 School of Civil and Transportation Engineering, Guangdong University of Technology, Guangzhou 510006, China; 13823893571@139.com; 4 School of Transportation and Civil Engineering and Architecture, Foshan University, Foshan 528225, China; guoyc@fosu.edu.cn

**Keywords:** PET-modified environmentally friendly high-ductility (P-EHDC), fracture performance, *J*-integral method

## Abstract

The utilization of polyethylene terephthalate (PET) powder as aggregate in the development of environmentally friendly high-ductility composites (P-EHDC) offers a promising pathway for advancing sustainable and high-performance concrete materials. Despite its potential, the fracture behavior of P-EHDC—particularly under the influence of alkali-activated precursors—remains insufficiently explored. In this study, the fracture performance of P-EHDC was evaluated by varying the precursor composition ratios (GGBS:FA = 4:6, 3:7, and 2:8) and PET powder replacement ratios (0%, 15%, 30%, and 45% by volume). Fracture modes, Mode I fracture energy (*G*_F_), and crack propagation behavior were analyzed using the *J*-integral method. All specimens exhibited ductile fracture characteristics, a clear contrast to the brittle failure observed in conventional concrete. The replacement of 15 vol% PET powder significantly increased *G*_F_ in precursor systems with higher GGBS content (4:6 and 3:7), and 30 vol% was more effective in fly ash-rich systems (2:8). The *J*-integral method, which offers broader applicability compared to conventional methods such as the double-K fracture model, provided a more comprehensive understanding of the fracture behavior. The results showed that PET powder reduced the matrix fracture toughness, promoted matrix cracking, and weakened the fiber-bridging effect, leading to enhanced energy absorption via fiber pull-out. At low PET powder replacement ratios (e.g., 15 vol%), the cracking threshold of the matrix was not significantly reduced, while more fibers engaged during the crack instability stage to absorb fracture energy through pull-out. This behavior highlights the synergistic toughening effect between PET powder and fibers in the P-EHDC system. The effect became more pronounced when the PET content was below 45 vol% and the precursor matrix contained a higher proportion of GGBS, leading to enhanced ductility. This study introduces a novel approach to fracture behavior analysis in PET-modified alkali-activated composites and provides theoretical support for the toughening design of high-performance, low-carbon concrete materials.

## 1. Introduction

High-ductility concrete (HDC), such as engineered cementitious composites (ECC) [1,2,3], aims to address the inherent brittleness of traditional concrete. Through micromechanical mechanisms governing the interaction between the cementitious matrix and fibers, HDC demonstrates significant tensile strain-hardening behavior and multiple cracking characteristics [4,5,6], enhancing material toughness. ECC has gained attention for its high ductility and crack control capabilities [7,8,9,10].

The production of ECC typically requires large amounts of traditional cementitious materials, such as Portland cement. However, in light of the increasing global climate challenges, countries worldwide are setting carbon reduction goals. China has specifically committed to reaching peak carbon emissions by 2030 and achieving carbon neutrality by 2060. The production of Portland cement generates harmful substances, including CO_2_, nitrogen oxides, sulfur oxides, and dust. For every ton of cement produced, 0.7 to 1.1 tons of CO_2_ are emitted, contributing to 5–7% of global carbon emissions annually [11,12,13,14,15]. As a result, developing alternative green binders has become a research focus. Alkali-activated composites, which use industrial waste materials like slag (GGBS) and fly ash (FA) with volcanic ash activity, combined with alkaline activators, are considered promising green binders [16,17,18]. Compared to Portland cement, alkali-activated materials reduce CO_2_ emissions by 55–75% and energy consumption by 36%, while offering superior properties like high compressive strength and low permeability [19,20,21,22,23]. Thus, these materials are seen as promising substitutes for Portland cement [24,25]. Alkali-activated materials are also viewed as a viable alternative for producing high-ductility alkali-activated concrete (HDAAC), marking a new direction for high-performance concrete development.

In the past few decades, plastic has accounted for approximately 13% of the global annual municipal solid waste (MSW), and 19.2% and 16.4% of the total MSW sent to landfills and incineration, respectively. However, plastic recycling accounts for only 4.4% [26]. The inadequate management and disposal of these plastics have led to severe soil and water pollution [27]. Polyethylene terephthalate (PET) is commonly used to manufacture plastic bottles and transparent packaging, which are discarded after single use. Studies have shown that using plastic waste as a replacement for natural aggregates in concrete can reduce the extraction of natural resources and mitigate the negative environmental impacts of plastic [28,29,30,31].

Existing research indicates that the application of waste plastic in cement-based composites presents multidimensional technical characteristics. In fiber-reinforced systems, PET fibers have been shown to effectively enhance the mechanical properties of engineered cementitious composites (ECC) and engineered geopolymer composites (EGC). Their reinforcement effect competes with conventional polyvinyl alcohol (PVA) and polypropylene (PP) fibers in terms of tensile strength, compressive strength, and multiple crack control [32]. Particularly, the interface bonding characteristics of PET fibers provide significant advantages in matrix stress transfer and crack propagation suppression. Studies on plastic as a substitute for aggregates show significant differences in the performance impact depending on the type of plastic. When PET and PE are used as fine aggregate replacements, both lead to a reduction in compressive, tensile, and flexural strengths, with PE having a more pronounced negative effect [33]. However, it is noteworthy that while the incorporation of plastic aggregates weakens strength parameters, it does not significantly deteriorate the material’s toughness, indicating its potential value in specific engineering scenarios. Mechanism studies suggest that the interfacial transition zone (ITZ) between plastic particles and the cement matrix is a key factor affecting material strength. When the plastic content exceeds a critical threshold, the significant reduction in interfacial bonding strength and adhesion becomes the primary limiting factor [34]. On the other hand, experiments replacing quartz powder with PET powder show an optimal replacement threshold. When the replacement ratio is controlled around 10%, the compressive and tensile strengths of geopolymer concrete can be increased by approximately 5.8% and 24%, respectively. This enhancement is attributed to the densification effect of PET powder on the matrix microstructure [35]. However, this enhancement effect is highly sensitive to the dosage, as excessive incorporation leads to performance degradation due to interfacial bonding deterioration. Scanning electron microscopy (SEM) analysis further reveals a negative correlation between plastic content and ITZ quality. A high content of plastic particles forms weak bonding regions at the matrix-plastic interface, thereby reducing the overall performance of the composite material [35].

The fracture behavior of conventional concrete is typically characterized by brittle or quasi-brittle fracture. Under the framework of fracture mechanics, its failure can be analyzed using linear elastic fracture mechanics methods [36,37,38], such as the traditional dual-K fracture criterion [39,40,41], as the double-K fracture model characterizes the fracture behavior of concrete by introducing two distinct fracture toughness values for crack initiation and unstable propagation. However, high-ductility concretes, such as HDAAC, exhibit distinct fracture characteristics compared to conventional fiber-reinforced concretes; they display tough fracture behavior and cannot satisfy the basic assumptions of the dual-K fracture model, specifically the linear superposition assumption [42,43]. As a result, some studies suggest that the *J*-integral method, based on nonlinear principles, is more suitable for assessing the fracture behavior of high-ductility concretes. The *J*-integral method’s path-independent nature avoids the issues associated with the linear superposition assumption [44].

In summary, current research on PET-modified concrete primarily focuses on concrete using Portland cement as the binder, and those studies mainly investigate basic mechanical properties such as compressive, tensile, and flexural strength, while research on fracture mechanics is relatively limited. Moreover, there is a significant lack of studies on the application of PET powder in alkali-activated high-ductility composites, which are designed using alkali-activated binders and synthetic fibers as reinforcement materials. On the other hand, conventional analytical models such as the double-K fracture model are only applicable to ordinary concrete materials exhibiting brittle failure.

Therefore, this study utilized PET powder to modify alkali-activated high-ductility composites, thereby developing PET-modified engineered high-ductility concrete (P-EHDC). Based on the *J*-integral fracture model, the study investigated the effects of different precursor material ratios (GGBS:FA = 4:6, 3:7, and 2:8) and various PET powder replacement ratios for quartz powder (0%, 15%, 30%, and 45% by volume) on the fracture behavior of P-EHDC. Through three-point bending tests, the failure modes of P-EHDC were analyzed, and the material’s performance was comprehensively evaluated from both localized crack propagation and overall deformation perspectives using *P-CMOD* and *P-δ* curves. The Model-I fracture energy (*G*_F_) and fracture energy calculated using the *J*-integral method were also determined, with a thorough analysis of the material’s energy dissipation capacity. Additionally, scanning electron microscopy (SEM) is employed for microstructural analysis.

## 2. Materials and Methods

### 2.1. Materials

**Figure 1:**
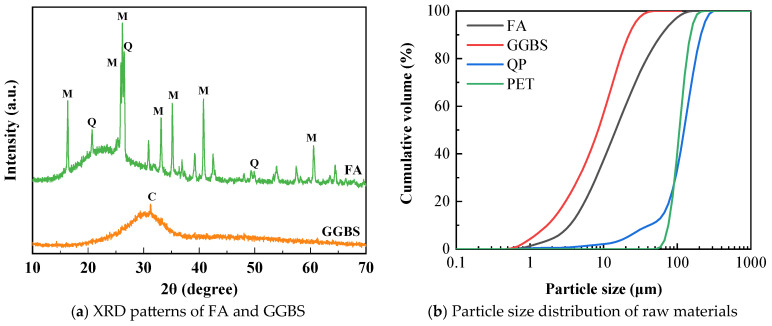
Physical and chemical property parameters of raw materials.

**Figure 2:**
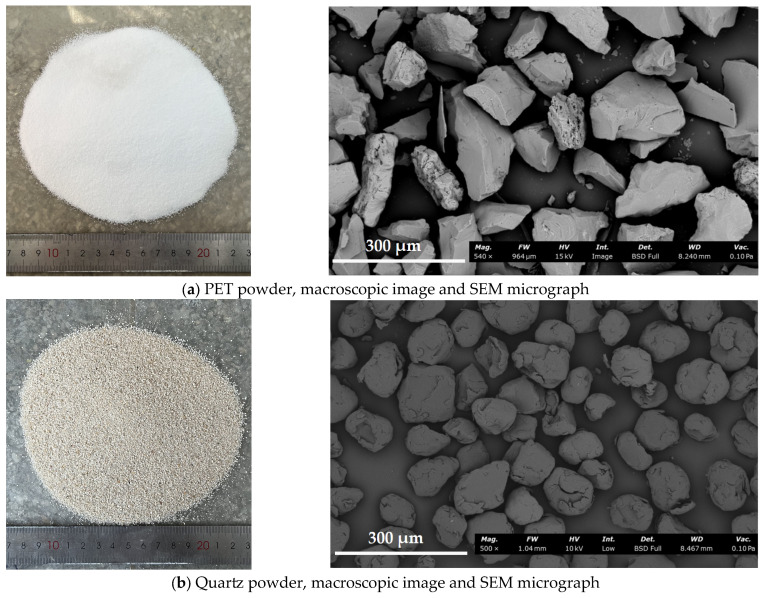
Appearance of aggregates.

The materials used in this study include ground granulated blast-furnace slag (GGBS), class F fly ash (FA), quartz powder (QP), plastic raw material polyethylene terephthalate powder (PET powder), alkali activators, ultra-high molecular weight polyethylene fibers (PE fibers), retarders (barium chloride), and water. The specific parameters of the materials used in this study are shown in Table 1 and Table 2 and Figure 1. GGBS, a white solid powder, was sourced from Longze Water Purification Materials Co., Ltd. (Gongyi, China). FA, a gray-black solid powder, was also sourced from Longze Water Purification Materials Co., Ltd. (Gongyi, China). GGBS and FA compose the binder material. PET powder, a white powder with a density of 1.68 × 10^3^ kg/cm^3^, was obtained from Guangyuan Plastic Co., Ltd. (Dongguan, China). Quartz powder was used with a particle size range of 76 μm to 150 μm and a density of 2.65 × 10^3^ kg/cm^3^. The appearance of the two aggregates is shown in Figure 2. Sodium silicate solution was sourced from Yourui Refractory Materials Co., Ltd. (Jiaxing, China). Sodium hydroxide, a white solid particle with a purity of 96%, was purchased from Xilong Science Co., Ltd. (Jiaxing, China). PE fibers, white bundled monofilaments, were provided by Suotite Special Line Co., Ltd. in (Dongguan, China). Barium chloride (BaCl_2_), used as a retarder to delay the initial setting time of P-EHDC, at a dosage of 1% by mass of the precursors, was sourced from Xilong Science Co., Ltd. (Jiaxing, China) [45]. The reaction between BaCl_2_ and sodium silicate solution can produce a dense cladding layer, thus preventing the GGBS from coming into direct contact with the sodium silicate solution and achieving a certain retarding effect [46].

The mix proportions used in this study are shown in Table 3. Three precursor matrix types, B1, B2, and B3, were prepared based on different GGBS and FA ratios. These ratios primarily affect the strength of the material matrix, with strength decreasing as the proportion of GGBS decreases [47,48]. Similarly, when determining the final proportions of GGBS and FA, primary consideration was given to the effect of precursor ratios on the matrix strength. Preliminary experiments were also conducted to guide the decision, resulting in the selection of three ratios as shown in Table 3: 4:6, 3:7, and 2:8. Based on these three different binder ratios, PET powder was added to replace quartz powder at volumes of 0%, 15%, 30%, and 45% by volume. Thus, this study involves 12 mix designs of P-EHDC, with a water-to-binder ratio (*w*/*b*) of 0.32 for all binder compositions.

**Figure 3:**
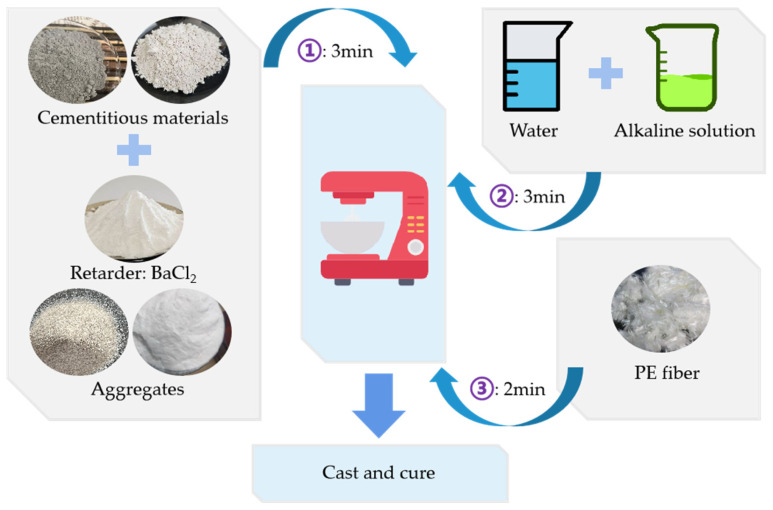
Preparation procedure for P-EHDC.

The specimen preparation process is divided into three main stages. First, a sodium hydroxide solution with a molarity of 14 mol/L is prepared using solid sodium hydroxide 24 h prior to casting. After allowing it to cool to room temperature naturally, the solution is mixed evenly with sodium silicate solution, then left to cool to room temperature again. Second, all dry materials, except for the fibers, are added to a planetary mixer as shown in Table 3 and mixed for 3 min to ensure uniform blending. The alkali activator and additional water are then mixed and added to the mixer, where the mixture is stirred for another 3 min. In the final stage, PE fibers are added, and the mixing is completed within 2 min, as shown in Figure 3. Finally, the fully mixed P-EHDC is poured into a prismatic mold. After initial setting, the specimens are covered with plastic film for curing. After 24 h, the specimens are removed from the molds and immersed in a water tank for continuous curing until 28 days [17].

**Figure 4:**
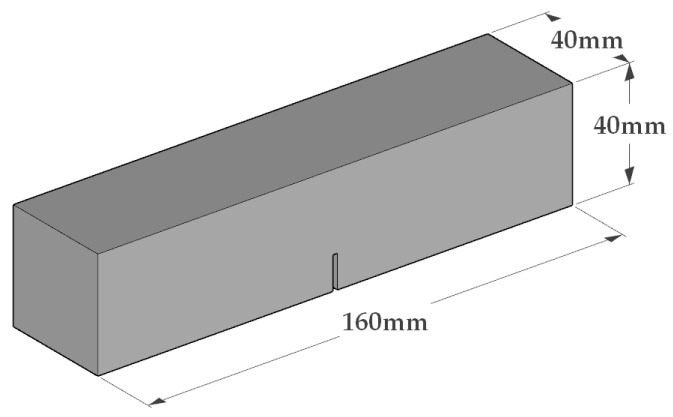
Schematic dimension of fracture specimen.

The schematic diagram of the fracture specimen used in this study is shown in Figure 4. The specimen dimensions are 40 mm × 40 mm × 160 mm, with a span of 120 mm. The preformed notch was manually cut with a cutting machine after the specimen had dried and completed curing. The notch depth is 16 mm, with a width within 1 mm, and the depth error is controlled within 0.5 mm.

### 2.2. Testing Setup

#### 2.2.1. Fracture Test

**Figure 5:**
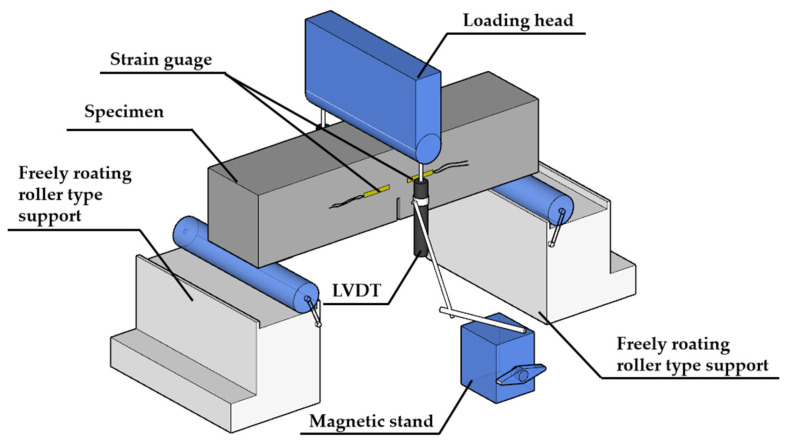
Schematic diagram of the fracture experimental setup.

The fracture testing setup used in this study is shown in Figure 5. Following the JCI-S-001 recommendation [49], a three-point bending fracture test was conducted to assess the fracture properties of concrete based on the *P-CMOD* curve. Referencing JCI-S-001 and existing research on the fracture performance of high-ductility concrete [36,42,44,50], a three-point bending test was employed to investigate the fracture behavior of P-EHDC. During the three-point bending loading process, the testing machine used a displacement control mode with a rate of 0.1 mm/min. Two displacement gauges with a range of 25 mm were placed on both sides of the specimen to measure the deflection at the midpoint. A clip-on extensometer was used at the bottom of the specimen to measure the crack opening displacement (*CMOD*). Additionally, the crack initiation load of P-EHDC was measured using two 10 mm strain gauges horizontally arranged at both ends of the preformed notch.

#### 2.2.2. SEM Analysis

The scanning electron microscopy was conducted using an S-3400N-II scanning electron microscope, produced by Hitachi (Tokyo, Japan). The SEM test mainly characterized the morphology of the interface between the aggregates and the matrix in the fractured cross-sections.

## 3. Results and Discussion

### 3.1. Failure Mode

**Figure 6:**
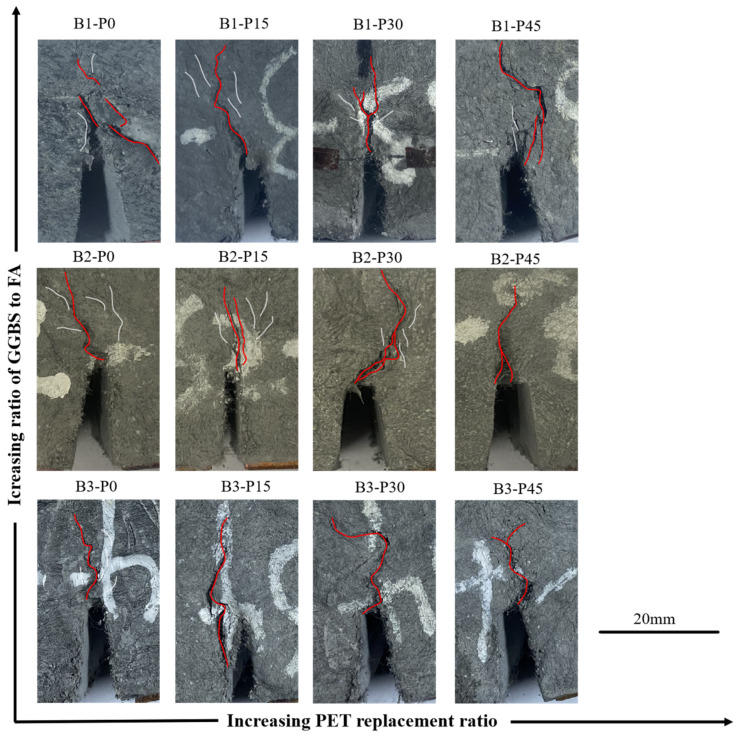
Failure mode of P-EHDC.

The typical failure mode of the P-EHDC fracture specimen is shown in Figure 6. During the three-point bending test, microcracks were first observed at the tip of the preformed notch, indicating that the applied load had reached the crack initiation load of P-EHDC. As the loading continued, macrocracks gradually propagated from the notch tip (refer to the red curve in Figure 6), accompanied by an increasing opening angle at the notch. Ultimately, the macrocracks continued to develop until the specimen became unstable. Fibers were progressively pulled out or fractured from the matrix, and the specimen lost all its load-bearing capacity. As shown in Figure 6, the change in PET powder replacement ratio affects the initiation of microcracks near the main crack of the specimen (refer to the white curve in Figure 6). For example, in the B1 and B2 series test groups, microcracks were still observable on the surface of specimens with 0 vol% and 15 vol% PET powder replacement. However, as the PET powder replacement ratio increased, microcracks were no longer visible. On the other hand, the increase in PET powder replacement ratio made the propagation path of the main crack more tortuous, exhibiting the so-called “ductile fracture” mode. The change in failure mode indicates that replacing quartz powder with PET powder as aggregate increased the fracture surface area. Furthermore, PET replacement altered the matrix properties, and the tortuous main crack path suggests unevenly distributed defects in the matrix. The figure also shows that the reduction in matrix strength, as indicated by the GGBS-to-FA ratio, caused the macrocrack propagation path to become more distorted.

### 3.2. P-δ Curves and P-CMOD Curves

**Figure 7:**
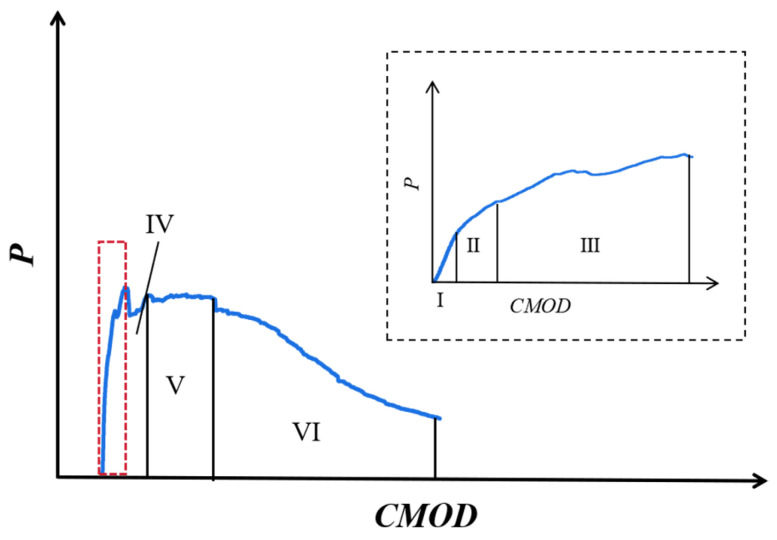
Schematic diagram of the stages in the *P-CMOD* curve for ductile fracture materials.

**Figure 8:**
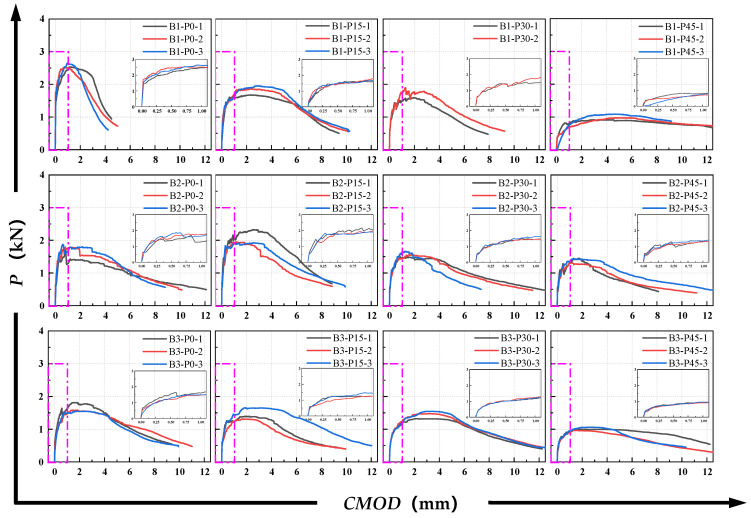
*P-CMOD* curves of P-EDHC.

**Figure 9:**
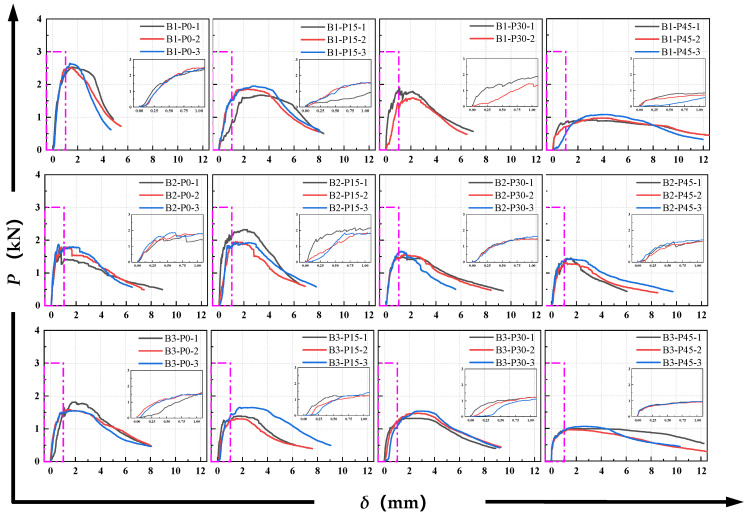
*P-δ* curves of P-EDHC.

Figure 7 shows the typical *P-CMOD* curve of ductile materials. The P-δ and *P-CMOD* curves for all P-EHDC specimens are shown in Figure 8 and Figure 9. It is observed that the curve propagation process shares common characteristics across all test groups. During the initial loading phase, the load increases rapidly at a nearly constant rate. After reaching the first inflection point, the load develops to the first peak value, then fluctuates around the peak load, forming a noticeable platform segment. Based on the fracture failure mode analysis, the appearance of the platform segment coincides with the time when the main crack in the fracture surface develops tortuously and when numerous microcracks form. More specifically, the *P-CMOD* curves for P-EHDC specimens exhibiting “ductile fracture” go through the following six stages, is also shown in Figure 7.

Stage I is the linear elastic stage, where the load is primarily carried by the P-EHDC matrix. In this stage, PE fibers exist as embedded defects within the matrix, which is due to the significant difference in elastic modulus between the PE fibers and the matrix [51,52]. Stage II is the inelastic deformation stage, where the load reaches the crack initiation load, and microcracks begin to form at the edges of the preformed notch. In Stage III, the load continues to increase with minor fluctuations. Notably, the slope of the curve decreases in the first three stages, meaning the rate of load increase slows down, and the stiffness of the P-EHDC decreases. In Stage IV, the *P-CMOD* curve begins to show noticeable oscillations, with the load continuing to increase amidst the fluctuations and accompanied by the formation of new cracks. This indicates that the specimen has entered the multi-crack stable propagation stage. In Stage V, a small platform section appears on the curve, with the load no longer increasing significantly. Although some microcracks still form at the notch tip, the matrix is almost no longer active, and the PE fibers begin to carry the majority of the load. This signifies that the width of the most prominent main crack at the notch is continuously increasing, and the specimen is about to enter the instability stage. Stage VI is the crack instability and propagation stage, where fibers begin to pull out or fracture, and the load drops significantly, accompanied by rapid expansion of the main crack, ultimately leading to specimen failure.

From Figure 8 and Figure 9, it is evident that, compared to B1-P0, B2-P0, and B3-P0, the slope of Stage I in the *P-CMOD* curve significantly decreased as the precursor matrix of P-EHDC changed. This indicates that the reduction in the GGBS ratio in the precursor weakens the stiffness of the linear elastic stage of P-EHDC. Since the load is primarily carried by the P-EHDC matrix in this stage, it suggests that the alkali-activated binder system with GGBS and FA as precursors shows a positive effect of GGBS on the strength of the cementitious products, and this is consistent with existing research findings [47,48]. On the other hand, as the PET replacement ratio increased, the curves of the first three stages in the *P-CMOD* curve become more gradual, with the slope significantly decreasing. The crack initiation load at the end of Stage II also gradually decreases. Moreover, the peak load that the specimen can reach decreased as the PET content increased.

### 3.3. Mode I Fracture Energy

The fracture energy G_F_ of concrete is one of the key parameters for evaluating its fracture performance and energy dissipation capacity. Although there are some differences in the definition and measurement methods of fracture energy in different standards and codes, in this study, despite the formation of some microcracks during the fracture process of P-EHDC specimens, the primary crack still plays a crucial role in determining the fracture energy of the specimen. Therefore, the G_F_ of P-EHDC is measured based on the JCI-S-001-2003 standard. According to the definition in the JCI-S-001-2003 standard, fracture energy G_F_ refers to the energy absorbed by the specimen per unit area when a crack is formed along a pre-set path. The specific calculation formulas can be referred to in Equations (1) and (2).

(1) $$G_{F}=\frac{0.75W_{0}+W_{1}}{A_{lig}}$$

(2) $$W_{1}=0.75\left(\frac{S}{L}m_{1}+2m_{2}\right)g\cdot {CMOD}_{0}$$

(3) $$A_{lig}=B(W-a_{0})$$

where *W*_0_ represents the area under the *P-CMOD* curve; *W*_1_ is the work done by the self-weight of the specimen and the fixtures; *A*_lig_ is the ligament area; *B*, *W*, and *a*_0_ are the specimen’s thickness, height, and notch depth, respectively; *S*/*L* is the ratio of the span to the total length of the specimen; *m*_1_ and *m*_2_ are the weights of the specimen and the fixtures not fully fixed in the test machine, with *m*_2_ typically taken as 0; g = 9.81 m/s^2^ is the acceleration due to gravity; and *CMOD*_0_ is the *CMOD* value corresponding to 30% of the maximum load P_max_ on the *P-CMOD* curve.

**Figure 10:**
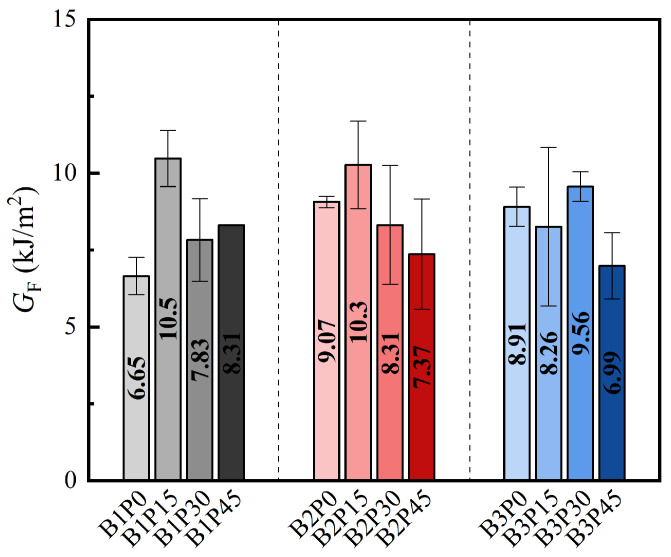
Effect of precursor matrix and PET powder replacement ratio on fracture energy.

Table 4 presents the results of the Model I fracture energy calculations, and Figure 10 shows the variation in fracture energy with the PET powder replacement ratio and precursor ratio. It is evident that the incorporation of PET powder has a positive impact on fracture energy. For instance, compared to B1P0, the fracture energy of B1P15, B1P30, and B1P45 increased by 60.3%, 19.5%, and 25.0%, respectively. Similarly, in the B2 and B3 series, the incorporation of PET powder also resulted in increased Model I fracture energy. This can be explained as follows: First, the alkali-activated high-ductility concrete made with GGBS and FA as precursors already exhibits ductile fracture characteristics [53,54], with microcracks surrounding the main crack. Introducing materials with hydrophobic properties or lower elastic moduli reduces the strength of the matrix and facilitates crack propagation [50], with PET powder serving this function. Under the influence of PET powder, the main crack path in P-EHDC becomes more tortuous, extending the *P-CMOD* curve. Even though the peak fracture load significantly decreases, the fracture energy is still enhanced.

### 3.4. Fracture Energy Based on J Integral Method

**Figure 11:**
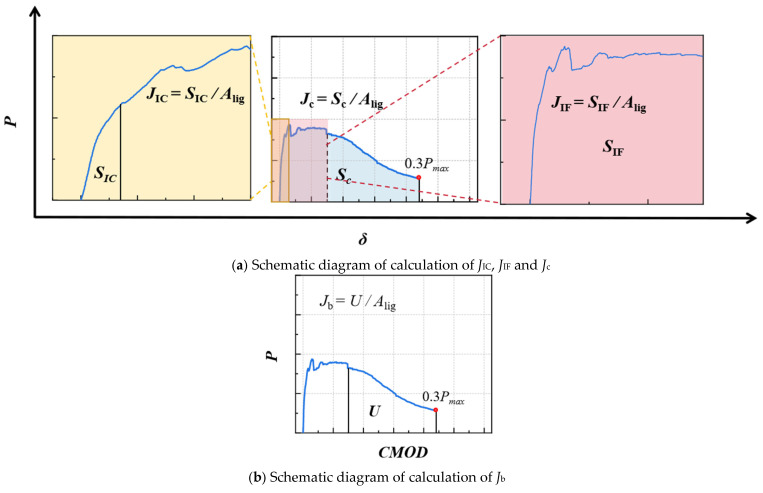
Schematic diagram of calculation of fracture energy based on *J*-integral method.

Various methods are used in the industry to evaluate the fracture behavior of materials, such as the dual-K criterion [39,40,41]. However, the dual-K criterion can only be applied when the fracture behavior of the material satisfies the important assumption of linear elastic fracture mechanics (as shown in Figure 11). Clearly, the ductile fracture behavior exhibited by P-EHDC in this study does not meet this assumption. Therefore, following the approach of Liu et al. [44] for assessing the fracture performance of strain-hardening concrete, this study employs a *J*-integral-based method to evaluate the ductile fracture behavior of P-EHDC. Based on previous research [38,43], this study introduces *J*_IC_, defined as the energy flowing into the plastic zone when macrocracks appear, which can be considered the material’s crack initiation fracture energy. Additionally, *J*_IF_ is introduced, defined as the energy localized in the plastic zone at the crack tip, marking the point of stable crack growth. The specific relationships are as follows:

*J* = *J*_IC_, The formation of first macroscopic crack,

*J_IC_* < *J* < *J*_IF_, The stage of stable crack growth,

*J* = *J*_IF_, Termination point of stable crack growth,

*J* > *J*_IF_, Unstable crack propagation stage of the material.

*J*_IC_ and *J*_IF_ are calculated by Equations (4) and (5):

(4) $$J_{IC}=\frac{2S_{IC}}{A_{lig}}$$

(5) $$J_{IF}=\frac{2S_{IF}}{A_{lig}}$$

where *S*_IC_ and *S*_IF_ represent the areas under the *P-δ* curve of P-EHDC from the origin to the crack initiation load *P*_ini_, and from the origin to the failure load *P*_IF_, respectively.

Given that P-EHDC is essentially a composite material—particularly due to the significant difference in properties between its fiber and matrix components—this study introduces a *J*-integral-based fracture energy analysis method to better investigate the respective contributions of the matrix and fibers to fracture behavior and energy dissipation throughout the entire cracking process. This approach aims to provide deeper insights into the underlying toughening mechanisms and enrich the understanding of the material’s fracture characteristics.

Fiber-reinforced high-ductility composites can transfer stress at the crack tip to the surrounding area through fibers, leading to the propagation of microcracks. As a result, the total energy absorbed during fracture *J* consists of two parts: the energy from the fiber pull-out process on the main fracture surface (fiber-bridging fracture energy, *J*_b_) and the energy from the microcracking process (energy associated with crack surface out-of-plane fracture, *J*_m_) [55]. To evaluate the toughening mechanism of PET powder in P-EHDC, this study calculates the composite total fracture energy *J*_c_, the total out-of-plane fracture energy *J*_m_, and the energy consumed by fiber bridging within the crack surface *J*_b_. Figure 11 provides a schematic of the fracture energy calculations based on the *J*-integral. It is particularly noted that, due to the good ductility of P-EHDC, it is difficult to load the specimen to complete fracture (i.e., to zero load). Therefore, 0.3P_max_ is used as the calculation endpoint, which is consistent with the calculation rule for Mode I fracture energy *G*_F_. The relationships and calculation methods for the composite total fracture energy *J*_c_, total out-of-plane fracture energy *J*_m_, and energy consumed by fiber bridging within the crack surface *J*_b_ are as follows: [55]

(6) $$J_{c}=J_{m}+J_{b}$$

(7) $$J_{c}=\frac{2S_{c}}{A_{lig}}$$

(8) $$J_{b}=\frac{U}{A_{lig}}$$

The determination of Equation (4) is based on the research conducted by the ASTM-24 committee [44,56] on three-point bending fracture specimens, where *S*_c_ represents the area under the *P-δ* curve of the specimen. Equation (5) calculates the fracture energy *J*_b_ consumed by fiber bridging using the post-peak area *U* from the *P-CMOD* curve.

**Figure 12:**
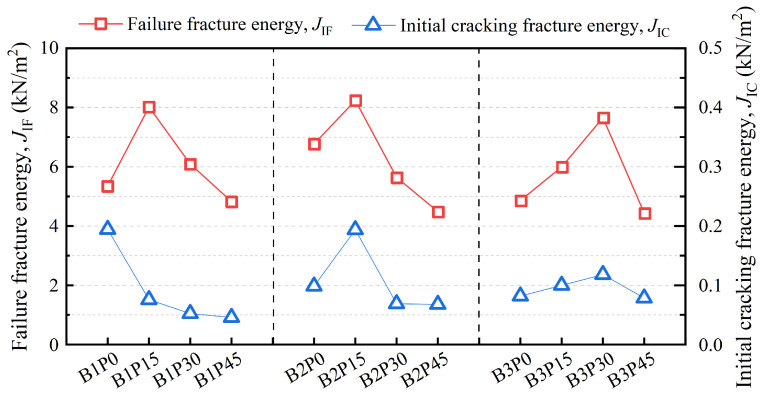
Effect of precursor matrix and PET powder replacement ratio on *J_IC_* and *J_IF_*.

Figure 12 and Table 5 present the variation of *J*_IC_ and *J*_IF_ for P-EHDC calculated based on the *J*-integral method, along with the specific data. As shown in Figure 12, the crack initiation fracture energy *J*_IC_ is relatively small compared to the failure fracture energy *J*_IF_ for all specimen groups, indicating that the first stage of fracture behavior in all groups was not fully developed. As the PET powder content increases, the overall trend for *J*_IC_ in the B1 series is a decrease. However, for the B2 and B3 series, the *J*_IC_ values initially increase and then decrease, with the peak values observed for B2P15 and B3P30. Notably, B2P15 exhibits the highest *J*_IC_ among all test groups with the same precursor ratio and PET powder replacement ratio, reaching 0.194 kN/m^2^, which is similar to B1P0. The failure fracture energy *J*_IF_ is similar to the Mode I fracture energy, as both are determined by the area under the *P-δ* curve. In this study, *J*_IF_ shows a significant response to PET powder replacement rates. A small amount of PET powder increases *J*_IF_, as seen in B1P15, B2P15, and B3P30. However, when the PET powder replacement ratio exceeds 30%, *J*_IF_ decreases significantly. Specifically, the lowest *J*_IF_ values across different precursor ratio groups are observed at a 45 vol% PET powder replacement ratio, with values of 4.83 kN/m^2^, 4.49 kN/m^2^, and 4.44 kN/m^2^, respectively, representing reductions of 39.8%, 45.4%, and 42.0% compared to the failure fracture energies of B1P15, B2P15, and B3P30. On the other hand, changes in matrix strength affected the trend of *J*_IF_ variation. In groups with different matrix strengths, the specimen with the highest *J*_IF_ does not have the same PET powder replacement ratio. The highest *J*_IF_ = 8.24 kN/m^2^ was also observed in B2P15.

*J*_IC_ and *J*_IF_ represent the initiation and failure critical points of P-EHDC, respectively. In Figure 12, the curve of the initiation fracture energy *J*_IC_ shows visible fluctuations, indicating that increasing the PET powder replacement ratio to 45 vol% can reduce the initiation fracture energy *J*_IC_ to a lower level. On the other hand, changing the GGBS content in the precursor (from B1 to B3) at the same PET powder replacement ratio does not show a clear trend regarding its impact on *J*_IC_. From the values and the range of change in *J*_IC_, it can be seen that lower GGBS content and higher PET powder content are detrimental to the development of *J*_IC_. Su et al. [38,52] pointed out that the load before cracking is primarily borne by the matrix in fiber-reinforced concrete. Therefore, a low GGBS content and high PET powder content in the precursor are not conducive to the crack initiation strength of the matrix. Carpinteri et al. [57] indicated that the role of fibers in fiber-reinforced concrete is to bridge cracks in the matrix, preventing crack propagation until fiber debonding, pullout, or failure occurs. The mechanism of fiber action in P-EHDC is similar in this regard.

On the other hand, the failure fracture energy *J*_IF_ is considered to represent the endpoint of crack stable development and the onset of material failure. Moreover, the level of *J*_IF_ reflects the energy borne by fibers in bridging cracks effectively in the matrix. The trend shown in Figure 12 indicates that *J*_IF_ initially increases and then decreases as the PET powder replacement ratio increases. Hou et al. [58] pointed out that rubber particles not only reduce the toughness of the matrix but also play a bridging role to some extent. The synergistic effect of both factors leads to a reduction in the crack width of HDC. The role of PET powder should be similar to that of rubber particles. The increase in *J*_IF_ with PET powder substitution is mainly due to its own bridging effect and the creation of more microcracks as the matrix fracture toughness decreases. This also helps fibers bridge more effectively and absorb more energy. However, this positive synergistic effect is limited to a certain range of PET powder replacement ratios, which, in this study, ranges from 15 vol% to 30 vol%.

**Figure 13:**
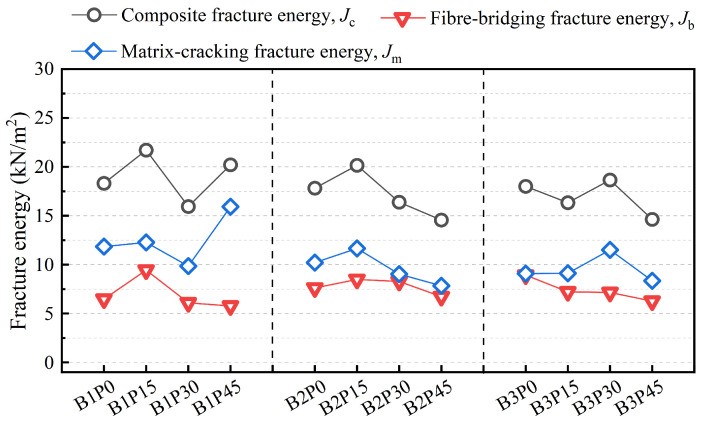
Effect of precursor matrix and PET powder replacement ratio on *J_m_*, *J_b_* and *J_c._*

Figure 13 shows the effect of PET powder substitution ratio on *J_m_*, *J_b_*, and *J_c_* in different precursor matrices. According to the calculation assumptions of the *J*-integral and the method presented in Equation (7), *J_c_* represents the critical point of P-EHDC’s final failure. It includes the total fracture energy from the first four stages of P-EHDC’s fracture behavior and the energy consumed during crack instability and development. It can be directly observed that the trend of *J_c_* across all test groups is consistent with the trend of *G*_F_ shown in Figure 10. The highest values of *J_c_* in the three precursor series were found at PET powder replacement ratios of 15 vol%, 15 vol%, and 30 vol%, with values of 21.7 kN/m^2^, 20.2 kN/m^2^, and 18.7 kN/m^2^, respectively. On the other hand, the *J_c_* variation in the B1 and B3 series exhibited two peaks, which is different from the B2 series. The lowest values of the composite fracture energy *J_c_* for each series were found in B1P30, B2P45, and B3P45, at 15.9 kN/m^2^, 14.6 kN/m^2^, and 14.6 kN/m^2^, respectively. These are 26.7%, 25.8%, and 21.9% lower than the highest values. Moreover, the change in precursor ratio did not significantly affect the composite fracture energy *J_c_* in the B1 and B3 series.

*J_b_* and *J_m_* represent the energy consumed by fiber bridging within the crack surface and the total fracture energy outside the crack surface, respectively. *J_b_* is calculated using Equation (8) and is primarily related to the crack instability and expansion phase. As shown in Figure 13, the energy consumed by fiber bridging within the crack surface (*J_b_*) is the smallest among the three physical quantities. In the B1 and B2 series, *J_b_* initially increases and then decreases with the increase in PET powder replacement ratio, while in the B3 series, *J_b_* decreases progressively. The variation of total fracture energy outside the crack surface (*J_m_*) with the PET powder replacement ratio follows the same trend as the composite fracture energy *J_c_*.

The calculated results for *J_b_* above indicate that when the precursor ratio of GGBS:FA is 4:6 and 3:7, a 15 vol% PET powder replacement enhances the energy dissipated by fiber pull-out and fracture within the crack surface. The energy consumed by fiber bridging (*J_b_*) is related to the bond between the fibers and the matrix. The bonding condition influences whether the fiber will pull out or fracture during the failure process. Fibers that pull out from the matrix dissipate more energy than those that fracture. In the B1 series, the B1P0 specimen, which contains no PET powder, has a more compact matrix interface. When the specimen enters stage VI of the fracture behavior phase, most of the fibers fail due to strong bonding with the matrix, as evidenced by the steep slope of the descending section of the *P-CMOD* curve in Figure 8. After replacing quartz powder with 15 vol% PET powder, the surface energy of the PET powder (40–50 mN/m) is lower than the surface tension of water (72 mN/m), and the hydrophobic properties of PET powder weaken the compactness of the matrix interface. This reduces the bonding between the matrix and fibers, allowing more fibers to pull out from the matrix.

However, in the B3 series, the low GGBS content in the precursor results in a weak matrix [47,48]. Even without PET powder (0 vol%), the matrix cannot form enough fiber bridging. As a result, *J_b_* decreases with increasing PET powder substitution. On the other hand, the incorporation of PET powder makes the matrix more prone to cracking. When the fibers can no longer absorb more fracture energy within the crack surface, the easily cracked matrix contributes to an increase in *J_m_*, which in turn enhances the composite fracture energy. This is reflected in the decrease of *J_b_* and the increase of *J_m_* in both B1P45 and B3P30, leading to an overall increase in *J_c_*. Similar findings were also supported by Su et al. [38] in their study of hydrophobic aggregates. Similarly, the reduction in matrix strength also plays a similar role.

### 3.5. The Analysis of Microstructure

**Figure 14:**
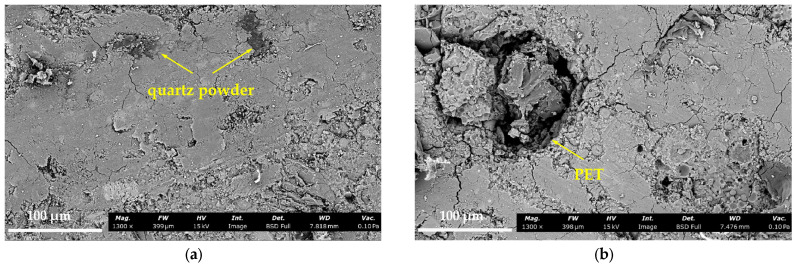
Microstructure of P-EHDC: (**a**) microstructure without incorporation of the PET powder; (**b**) microstructure with incorporation of the PET powder.

Considering the significant effect of PET powder on the fracture energy of P-EHDC, Figure 14 presents SEM images of the microstructure at the interface between the aggregate and the matrix for both PET powder-free and PET powder-incorporated P-EHDC materials, focusing on the interface density. From Figure 14a, it is evident that the boundary between the quartz powder particles and the surrounding matrix is rather indistinct, indicating a dense bond between them. In contrast, Figure 14b clearly shows an obvious transition zone between the PET particles and the surrounding matrix, resembling a trench. This further suggests the presence of a distinct interfacial transition zone (ITZ) between the PET powder and the cement matrix, which appears more readily identifiable at the same magnification level than that between QP and the matrix. This phenomenon occurs because PET, as a hydrophobic material, naturally creates a less cohesive interface with the matrix. Notably, by comparing the overall interface density of the materials at the same observation scale, it is clear that the interface in the PET-free specimen is much denser. In Figure 14b, numerous fine cracks can be observed around the PET particles in the matrix, indicating that the incorporation of PET weakens the matrix strength and promotes cracking. This observation helps explain the increase in Jm with higher PET powder replacement rates, as seen in B1P45 and B3P30 in Section 3.4. Even at relatively low levels, PET powder can enhance the synergy with fibers, contributing to the material’s toughness.

## 4. Conclusions

This study experimentally investigated the fracture behavior of P-EHDC under different precursor ratios and PET powder replacement ratios. The Mode I fracture energy was calculated to evaluate its energy absorption capacity, and the double-*J* fracture criterion was used to calculate *J*_IC_ and *J*_IF_ for assessing the fracture performance of P-EHDC. Based on the *J*-integral method, the total composite fracture energy *J_c_*, the energy consumed by fiber bridging within the crack *J_b_*, and the total fracture energy outside the crack *J_m_* were calculated. Additionally, through microstructural observations, the influence mechanisms of precursor ratio and PET powder replacement ratio on the fracture performance of P-EHDC were comprehensively studied, along with the toughening principles. The following conclusions were drawn:

(1) All P-EHDC specimens in this study exhibited “ductile fracture” behavior, with the final failure mode characterized by a main crack accompanied by some microcracks. The increase in PET powder replacement ratio led to a more tortuous path for the main crack but also reduced the initiation of surrounding microcracks to some extent. PET replacement altered the matrix properties, and the tortuous main crack path suggests unevenly distributed defects in the matrix. This should not be regarded as a negative outcome, as the tortuous crack path of the matrix suggests greater fracture energy absorption.
(2) Compared to the precursor ratio, the PET powder replacement ratio has a more significant effect on Mode I fracture energy (*G*_F_). The presence of PET powder promotes cracking in the material, leading to the formation of more fine microcracks around the main crack. The reduction in matrix strength has the same effect, this is also the reason why the main crack path propagates more tortuously.
(3) The hydrophobic PET powder weakens the overall density of the matrix, promoting material cracking. While PET powder itself has a certain bridging effect, excessive usage can still weaken the fiber-matrix bridging. The proportion of GGBS in the precursor affects the matrix strength—higher matrix strength tends to anchor fibers, leading to fiber breakage, while lower matrix strength allows more fibers to pull out and absorb fracture energy. In the B1 series, with a high GGBS proportion, replacing 15 vol% of quartz powder with PET powder lowers the crack initiation fracture energy (*J*_IC_). However, the PET powder’s bridging effect and reduced fracture toughness create more microcracks, which increases the fracture energy at failure (*J*_IF_). Despite the relatively low replacement ratio, the matrix’s crack initiation threshold does not significantly decrease, but it allows more fibers to pull out and absorb fracture energy during the crack instability phase. Therefore, compared to B1P0, B1P15 shows a 45.7% increase in *J_b_* and a 4.24% increase in *J_m_*.
(4) Based on the findings of this study, it is recommended to use a higher matrix strength with no more than 45 vol% PET powder as a replacement for quartz powder to prepare P-EHDC. This approach can improve the ductile fracture behavior and fracture energy of the material while enhancing the recycling of waste PET powder and promoting the green and low-carbon development of high-performance new materials. Admittedly, the study did not fully integrate the surface crack propagation process of the specimens with the *J*-integral-based fracture analysis. Future research could employ high-resolution imaging techniques to capture the detailed evolution of surface cracks, enabling correlation with fracture energy parameters derived from the *J*-integral method. This approach may contribute to the development of a more suitable framework for analyzing and evaluating the fracture behavior of high-ductility materials.

## Figures and Tables

**Table 1:** Property Parameters of cementitious materials and aggregate.

Property Parameters	CaO	SiO_2_	Al_2_O_3_	SO_3_	Fe_2_O_3_	MgO	TiO_2_	Other	Loss on Ignition (%)	Compressive Strength (MPa)	Elastic Modulus (GPa)
	wt%										
GGBS	34.0	34.5	17.7	1.64	1.03	6.01	/	5.12	0.840	/	/
FA	4.01	54.8	31.2	2.20	4.16	1.01	1.13	2.37	4.60	/	/
QP	0.09	89.2	5.97	0.23	0.67	0.09	0.20	0.65	/	/	/
PET	/	/	/	/	/	/	/	/	/	130	1.10

**Table 2:** Properties of PE.

Fiber Type	Length (mm)	Diameter (um)	Strength (MPa)	Aspect Ratio (L/d)	Density (g/cm^3^)	Elastic Modulus (GPa)	Elongation (%)
PE	12.0 (Mean value)	20.0	2500	600	0.970	120	3.70

**Table 3:** Mix design.

Cementitious Material ID	Mix ID	Mix Design (kg/m^3^)							
		GGBS	FA	QP	Alkaline Solution	Water	Retarder	PET	PE
B1	B1-P0	491	736	245	491	73.6	12.3	0.00	19.4
	B1-P15	491	736	209	491	73.6	12.3	23.3	19.4
	B1-P30	491	736	172	491	73.6	12.3	46.7	19.4
	B1-P45	491	736	135	491	73.6	12.3	70.0	19.4
B2	B2-P0	364	850	243	486	72.8	12.1	0.00	19.4
	B2-P15	364	850	206	486	72.8	12.1	23.1	19.4
	B2-P30	364	850	170	486	72.8	12.1	46.2	19.4
	B2-P45	364	850	134	486	72.8	12.1	69.3	19.4
B3	B2-P0	239	956	239	478	71.7	12.0	0.00	19.4
	B2-P15	239	956	203	478	71.7	12.0	22.7	19.4
	B2-P30	239	956	167	478	71.7	12.0	45.5	19.4
	B2-P45	239	956	132	478	71.7	12.0	68.2	19.4

Note: B1-P15 refers to a cementitious material with a GGBS to FA ratio of 4:6, and PET replacing 15% of the quartz powder by volume.

**Table 4:** Fracture energy calculation results.

Mix IDs	*Pini*	*Pmax*	*m*_1_	*CMOD*_0_	*W*_0_	*W*_1_	*G*_F_
	(kN)	(kN)	(kg)	(mm)	(kN·mm)	(kN·mm)	(kJ·m^−2^)
B1-P0	0.705 (0.022)	2.56 (0.025)	0.521 (0.001)	4.58 (0.39)	8.48 (0.77)	0.0244 (0.0022)	6.55 (0.61)
B1-P15	0.773 (0.015)	1.83 (0.171)	0.509 (0.001)	10.2 (0.5)	13.4 (1.2)	0.0376 (0.0033)	10.5 (0.9)
B1-P30	0.605 (0.025)	1.59 (0.60)	0.489 (0.007)	8.53 (0.91)	9.99 (1.72)	0.0270 (0.0046)	7.83 (1.35)
B1-P45	0.372 (0.025)	1.29 (0.77)	0.482 (0.003)	13.2 (0.1)	10.6 (0.0)	0.0282 (0.0000)	8.31 (0.00)
B2-P0	0.873 (0.101)	1.79 (0.02)	0.494 (0.006)	10.2 (1.6)	11.6 (0.23)	0.0316 (0.0006)	9.07 (0.18)
B2-P15	0.804 (0.110)	2.07 (0.33)	0.488 (0.001)	9.13 (0.67)	13.1 (1.8)	0.0353 (0.0049)	10.3 (1.4)
B2-P30	1.04 (0.09)	1.58 (0.06)	0.463 (0.002)	10.5 (2.8)	10.6 (2.5)	0.0271 (0.0063)	8.31 (1.93)
B2-P45	0.642 (0.044)	1.40 (0.14)	0.458 (0.002)	10.7 (2.5)	9.40 (2.30)	0.0238 (0.0058)	7.37 (1.80)
B3-P0	0.642 (0.044)	1.65 (0.83)	0.462 (0.004)	10.1 (0.8)	11.4 (0.8)	0.0291 (0.0021)	8.91 (0.63)
B3-P15	0.923 (0.031)	1.45 (0.50)	0.450 (0.011)	10.5 (1.3)	10.5 (3.3)	0.0261 (0.0082)	8.27 (2.58)
B3-P30	0.988 (0.022)	1.45 (0.30)	0.433 (0.003)	12.0 (0.3)	12.2 (0.6)	0.0292 (0.0015)	9.56 (0.48)
B3-P45	0.733 (0.009)	1.01 (0.19)	0.427 (0.002)	11.7 (1.2)	8.91 (1.38)	0.0210 (0.0032)	6.99 (1.08)

**Table 5:** Calculation results of fracture energy based on *J* integral method.

Mix IDs	*J_IC_* (kJ/m^2^)	*J_IF_* (kJ/m^2^)	*J_c_* (kJ/m^2^)	*J_b_* (kJ/m^2^)	*J_m_* (kJ/m^2^)
B1-P0	0.194 (0.041)	5.35 (0.99)	18.3 (1.6)	6.48 (0.68)	11.8 (0.9)
B1-P15	0.0757 (0.0455)	8.02 (1.19)	21.7 (1.1)	9.44 (0.99)	12.3 (0.9)
B1-P30	0.0524 (0.0031)	6.09 (1.22)	15.9 (2.5)	6.10 (1.61)	9.85 (0.88)
B1-P45	0.0456 (0.0209)	4.83 (0.088)	20.2 (1.6)	5.77 (0.15)	15.9 (2.4)
B2-P0	0.0985 (0.0476)	6.77 (1.88)	17.8 (0.6)	7.61 (1.40)	10.2 (0.9)
B2-P15	0.194 (0.068)	8.24 (2.32)	20.2 (2.3)	8.49 (0.69)	11.7 (2.0)
B2-P30	0.0689 (0.0217)	5.64 (1.63)	16.4 (3.7)	8.29 (0.15)	9.04 (2.14)
B2-P45	0.0677 (0.0107)	4.48 (0.82)	14.6 (3.6)	6.73 (1.43)	7.84 (2.21)
B3-P0	0.0816 (0.0180)	4.86 (1.32)	18.0 (0.8)	8.93 (1.15)	9.08 (1.09)
B3-P15	0.100 (0.034)	5.99 (1.81)	16.3 (4.8)	7.23 (2.11)	9.11 (2.11)
B3-P30	0.118 (0.022)	7.65 (0.18)	18.7 (1.0)	7.16 (0.80)	11.5 (0.3)
B3-P45	0.0783 (0.0301)	4.44 (1.16)	14.6 (2.2)	6.27 (0.48)	8.35 (2.43)

Note: the values are standard deviations in parentheses.

## Data Availability

The data presented in this study are available on request from the corresponding author. The data are not publicly available due to confidentiality issues.
